# Atlanta Society of Medicine

**Published:** 1899-10

**Authors:** 


### *SOCIETY REPORTS.*

### ATLANTA SOCIETY OF MEDICINE.

ATLANTA, GA., September 7, 1899.

*Regular Meeting of the Atlanta Society of Medicine.*

On account of the small attendance and at the request of the President, Dr. Dunbar Roy consented to postpone the reading of his paper until the next meeting of the society.

### REPORT OF CASES.

*Dr. W. L. Champion:* I wish to report a case of multiple chancre in a boy eighteen years of age. It is a unique case in the fact that one sore was genital and the other extra-genital, both appearing at the same time. One sore was on the glans penis and the other upon the upper lip. Outside of the location of the sores the case presented nothing of particular interest except the swelling of the glands about the throat, which was so severe as to interfere with deglutition for about a week. I waited, as I usually do, for the muscular eruption to appear before commencing constitutional treatment. The picture I show here is a very good one of the chancre on the lip. The day the picture was taken we could not get one of the sore on the penis on account of swelling which prevented retraction of the foreskin.

*Dr. Hardon:* I had a case recently which was a little out of the ordinary run and therefore probably of interest. The patient was pregnant and had been so since the 10th of November, which was the last menstruation. In January I was first called to see her and she was suffering then with abdominal pains and nervous symptoms, which I treated in a palliative way. That part of the case was not particularly interesting, as it yielded to treatment. She went on until the 26th of June and I was sent for, and when I reached the house the patient informed me that the amniotic fluid had escaped in a sudden gush at night and woke her up.

She had slight pains, but not enough to indicate labor. This continued for several days, with a slight additional escape of fluid, but still the labor did not come on. I watched her expecting the labor to begin. Finally the escape of the amniotic fluid ceased. I examined the abdomen and found that the womb had diminished in size so that the fundus was not above the level of the umbilicus. The walls were thin and the outline of the child was easily felt. The movements of the child continued and the fetal heart-beats continued strong and normal in strength. The patient continued this way day after day and week after week, and the slight pains at intervals keeping up as in the beginning of labor. This continued until the 19th of August, seven weeks and three days. In the meantime the fluid increased and the womb increased in size and reached the ensiform cartilage. I examined the fluid which had escaped but there was no odor of urine and the patient was sure it did not come from the bladder, and I was equally sure. Labor came on suddenly the 19th of August and the child was born after four expulsive pains, and it weighed only four and a quarter pounds. A reliable nurse was present and told me that the escape of amniotic fluid at the time was normal in amount. I do not know of a case on record where the fluid escaped and then began again after seven and a half weeks. The longest I have known from the rupture of the membranes to the birth of the child was two weeks. Therefore, from this point of view, the case was rather unique.

*Dr. Dunbar Roy:* The case mentioned by Dr. Champion brought to my mind a case I had some few weeks ago in a private patient, a young man of exemplary character. He came into the office and said his throat had been sore for the last few days and had trouble in swallowing. I looked into the mouth and saw typical mucous patches of the uvula on the side of the mouth. I inquired thoroughly into the case and he at first denied all possibility of infection, and his brother was in to see me the next day and told me he was sure that he had never had connection with a woman. However, on the next visit the young man told me that some six months ago he had connection with a woman, but so far as he knew never had the slightest abrasion or any ill effects in any way. To my mind, as I looked into the throat the appearance of the ulcers was typical, with the clean cut and reddish margin which is familiar to those who see a number of these, whether in the mouth or elsewhere. As he was clear in his history, I decided to give him no anti-syphilitic treatment, but to wait. I therefore cauterized these ulcers with nitrate of silver, thirty grains to the ounce, and in ten days they were healed. That was three weeks ago. I have kept tab on him and had him come to the office, but he has never had any eruption or roseola, and I do not think he will have any eruption. This was a case which looked like mucous patches, but I have come to find that it is a difficult thing to tell whether an ulceration in the mouth or nose is positively of syphilitic origin or not, because I have had so many cases that have yielded entirely to local applications and no systemic remedies given.

Some two weeks ago I had an old gentleman, about sixty years of age, who drank a good deal and had provocation for contracting this disease, and he had ulcers under his soft palate and yet no syphilitic remedies were given, but simply local stimulating applications were made, and he recovered. I had a young man with ulcers in the nose and had the appearance of syphilitic dyscrasia, and this healed also under local treatment. There was no enlargement of the glands and I have come to find out that there are a great many ulcers appearing in the mouth up under the soft palate, which are nothing more than catarrhal ulcers and have no record of systemic condition. Therefore, I am slow to say at the first or second or the third examination whether they are due to syphilis, because these patches can come from catarrhal conditions I am sure in my own mind, but this case was so typical in appearance that I would have sworn it was syphilitic. Yet he has recovered in three weeks and no enlargement of the glands, and whether there will be any in the future I do not know, but in my experience there will be no further eruption. I do not know whether you gentlemen who have treated these cases with mucous patches in the mouth have noticed these ulcers I speak of or not.

*Dr. J. L. Campbell:* I had quite an interesting case which is just convalescent now. A lady was delivered by a physician here in the city, and about three days later the lochia slowed up and then returned. She got up on the ninth day and went to work, and twelve days after confinement she had a chill and then the temperature went up and remained quite high with tongue dry and brown, and so one night about nine o'clock called me because could not get the other doctor, and a couple of days later got mad with the other doctor and would not allow him to come into the house, and he asked me to take charge of the case. He had been treating her for septicemia, and been washing out twice a day. Her pulse was 140 and temperature 104. She had some vaginitis, and I curetted to some extent and put in catheter and used the alcohol treatment as recommended by Dr. Noble. The temperature went down after two or three days. This alcohol treatment consists of injecting alcohol every hour to keep the dressings wet. The pulse came down to 120 and she developed what appeared to be a diphtheritic vaginitis. I removed the dressing and washed the vagina with one-half per cent. formalin. Her temperature came down to 99, and went along for three or four days and then went up to 103, but is now down to 99 and pulse 104. She was delirious and has considerable wandering now. I would like to know whether the delirium is due to the impression of the poison on the nervous system. During the relapse she had tenderness and pain in the lower limbs so that she could hardly move them, a sort of neuritis. This has almost passed away. She had a vesicular eruption on the body and had two or three abscesses on the neck, but these have now disappeared and she is on the good road to recovery, except the mind.

*Dr. Hardon:* What is the form of the delirium?

*Dr. Campbell:* She gets mad and wants to get out of bed; drove her husband out of the room; would not let her physician come about her, and thinks other people are mad with her.

*Dr. Roy:* What is the condition of her urine?

*Dr. Campbell:* She had albumen and casts in the urine; at first had almost suppression of urine, but not as much albumen last week as at first.

*Dr. Hardon:* There is a form of delirium which takes the form of delusional mania and which follows almost any form of septic infection. It can be the result of extreme alcoholic poisoning, appears sometimes where septic infection follows labor, but more commonly follows cases of operation with infection. In those cases the result is generally favorable. I have seen it last for five or six weeks and then the mind would clear up and recovery take place.

*Dr. Hood:* Dr. Campbell's case brought to mind a case of mine. A young widow came to my office with a severe case of vaginitis, and it seemed to me there was a diphtheritic deposit; had burning sensation and could not sleep. I had a bottle of petroleum emulsion sitting near, and I made a copious application of this, put in a piece of cotton and she got well. At the next visit all the diphtheritic deposit had disappeared.

*Dr. Champion:* As Dr. Roy mentions, I have seen these ulcers in the mouth without any history of syphilis or any symptoms of it whatever, that is, enlarged glands, eruptions, etc. I think that in Dr. Roy's case the eruption may have appeared before he saw it. I agree with Dr. Roy, that the stimulating application of the nitrate of silver will cause these ulcers to disappear. I use thirty grains to the ounce, to a saturated solution.

---

### TEST FOR MORPHINE IN MORPHINE HABITUÉS.

Collect about twenty ounces of urine from the suspected individual. If it has not an acid reaction, acidulate with dilute hydrochloric acid until blue litmus is reddened by it. Concentrate to about three ounces, and let it stand in a cool place for twelve hours, then filter. To the filtrate add sufficient sodium carbonate to render it alkaline; let it stand for twelve hours, filter and collect the precipitate, and wash this with distilled water made slightly alkaline with sodium carbonate, and dry. Digest the dried precipitate with pure alcohol at a gentle heat, and filter; evaporate the filtrate to dryness, dissolve the residue with dilute sulphuric acid, and test for morphine by the iodic-acid test, or other well-known tests. By this method morphine can be obtained, says the author, from persons taking but very minute amounts of the drug.—*Medical and Surgical Bulletin.*

